# Learning to improve medical decision making from imbalanced data without a priori cost

**DOI:** 10.1186/s12911-014-0111-9

**Published:** 2014-12-05

**Authors:** Xiang Wan, Jiming Liu, William K Cheung, Tiejun Tong

**Affiliations:** Department of Computer Science and Institute of Computational and Theoretical Studies, Hong Kong Baptist University, Kowloon Tong, Hong Kong; Department of Mathematics, Hong Kong Baptist University, Kowloon Tong, Hong Kong

**Keywords:** Medical decision making, Imbalanced data, Classification, Partial ranking

## Abstract

**Background:**

In a medical data set, data are commonly composed of a minority (positive or abnormal) group and a majority (negative or normal) group and the cost of misclassifying a minority sample as a majority sample is highly expensive. This is the so-called imbalanced classification problem. The traditional classification functions can be seriously affected by the skewed class distribution in the data. To deal with this problem, people often use a priori cost to adjust the learning process in the pursuit of optimal classification function. However, this priori cost is often unknown and hard to estimate in medical decision making.

**Methods:**

In this paper, we propose a new learning method, named RankCost, to classify imbalanced medical data without using a priori cost. Instead of focusing on improving the class-prediction accuracy, RankCost is to maximize the difference between the minority class and the majority class by using a scoring function, which translates the imbalanced classification problem into a partial ranking problem. The scoring function is learned via a non-parametric boosting algorithm.

**Results:**

We compare RankCost to several representative approaches on four medical data sets varying in size, imbalanced ratio, and dimension. The experimental results demonstrate that unlike the currently available methods that often perform unevenly with different priori costs, RankCost shows comparable performance in a consistent manner.

**Conclusions:**

It is a challenging task to learn an effective classification model based on imbalanced data in medical data analysis. The traditional approaches often use a priori cost to adjust the learning of the classification function. This work presents a novel approach, namely RankCost, for learning from medical imbalanced data sets without using a priori cost. The experimental results indicate that RankCost performs very well in imbalanced data classification and can be a useful method in real-world applications of medical decision making.

## Background

One of the challenging issues in medical data analysis is caused by the highly skewed proportion of differen sample types [1]. This often happens when one class of samples (positive or abnormal) is of limited size and sometimes difficult to collect while the other class (negative or normal) is much more abundant and much easier to find. Learning an effective classification model can be a difficult task if the data used to train the model are imbalanced. When samples of the majority class greatly outnumber samples of the minority class, the traditional classification models usually have a bias in favor of the majority class. This is because the goal of traditional classification modeling is to construct a function (or a classifier) based on the properties of training data so as to make as few errors as possible when being used to predict the class membership of new samples [2]. A range of classification methods, such as decision tree, neural network, nearest neighbor, logistic regression, and support vector machine, have been well developed. These methods, when applied to imbalanced medical data, will often produce high predictive accuracy over the majority class, but poor predictive accuracy over the minority class. Besides the medical data analysis, there are many other real world applications involving learning from imbalanced data, such as text classification [3,4], the fraudulent telephone call detection [5,6], oil spill detection [7], potential buyer selection in direct marketing [8], and etc. Nevertheless, the impact of this issue is particularly tremendous in medical data analysis because the cost of misclassifying a minority sample as a majority sample, e.g., patients miss the chance to be cured if they fail to be identified and diagnosed due to the wrong classification, is highly expensive and sometimes unaffordable.

There are three major approaches to dealing with imbalanced data sets, which are sampling, cost-sensitive learning, and boosting. The sampling approach is applied to create a more balanced class distribution in the training data by either over-sampling the minority class or under-sampling the majority class [4,9-13]. Both over-sampling and under-sampling have their benefits and drawbacks. They can be easily implemented and applied to all application domains with imbalanced data. But the classification performance can be very sensitive to the class ratio of the training data. One major drawback associated with over-sampling is that learning on duplicated samples can lead to overfitting [14]. On the other hand, under-sampling may result in the loss of information that comes with deleting samples [15].

While sampling approaches address the imbalanced learning problem at the data level, cost-sensitive learning methods target this problem at both the data level and the algorithm level [16]. Instead of creating balanced data distributions through different sampling strategies, cost-sensitive learning uses a cost matrix that describes the costs for misclassifying data samples [17-22]. The cost matrix encodes the penalty of misclassifying samples from one class as another. Some research works have provided the theoretical foundations of cost-sensitive methods in imbalanced learning problems [23,24] and various empirical studies have shown that cost-sensitive methods are superior to sampling methods in many application domains [25,26]. However, there is one major disadvantage of using cost-sensitive learning to handle the imbalanced medical data. It is that misclassification costs are often unknown and hard to estimate in medical decision making and the performance of cost-sensitive learning is very sensitive to different misclassification costs [26].

In contrast to sampling methods and cost-sensitive methods that are specially designed to address imbalanced learning problem, boosting is an off-the-shelf approach that is particularly effective in handling imbalanced data. The most common boosting algorithm is AdaBoost [27], which iteratively builds an ensemble of models with weighted samples. During each iteration, incorrectly classified samples are given high weights so that they will have high chance to be correctly classified in the next iteration. In the imbalanced classification, it is most likely that the minority class samples are misclassified at the beginning and naturally given higher weights in subsequent iterations. AdaBoost is particularly suitable for medical decision making since it does not require a priori cost. However, AdaBoost is still an accuracy-oriented algorithm and its learning process may still bias toward the majority class because samples in the majority class contribute more to the overall classification accuracy. As a result, the empirical study [16] shows that cost-sensitive methods outperform AdaBoost.

In this work, we present a novel boosting algorithm for the classification of imbalanced data. Instead of focusing on improving the class-prediction accuracy, our approach is to maximize the difference between the minority class and the majority class by using a scoring function. Intuitively, the basic idea is to translate the imbalanced classification problem into a partial ranking problem. In this partial ranking problem, we shall find a scoring (or ranking) function that can assign samples in the minority class higher scores than samples in the majority class or vice versa. Therefore, the target of our approach is to infer the pairwise relationship between samples in two classes. Compared to the cost-sensitive learning that explicitly uses cost matrix to learn a biased classifier toward the minority class, our method naturally embeds the importance of identifying minority samples in the new formulation and the relative importance between two classes is automatically learned from the data without using any priori knowledge.

## Methods

Given a sequence of *n* samples 〈(*x*_1_,*y*_1_),⋯,(*x*_*n*_,*y*_*n*_)〉 with labels *y*_*i*_∈{−1,1}, the boosting algorithm AdaBoost is equivalent to a forward stage-wise additive method using the exponential loss function

(1) $$ L(y, f(x)) = \exp(-yf(x)),  $$

where *f*(*x*) is a linear combination of multiple classifiers [28]. The loss function measures the difference between estimated and true values for an instance of data. To minimize this loss function, AdaBoost iteratively builds an additive model with an ensemble of classifiers where subsequent classifiers are learned in favor of those instances misclassified by previous classifiers. Therefore, in AdaBoost, the samples in the minority class that are often misclassified at start will be given higher weights in subsequent classifiers and then have higher chance to be correctly classified. Nevertheless, the loss function in Eq. (1) is defined on the overall prediction accuracy. Thus AdaBoost may still favor the majority class as it has higher impact in the loss function. Some cost-sensitive learning methods, such as AdaC1, AdaC2, AdaC3 [16], and AdaCost [21], extend AdaBoost with the pre-specified cost matrix, which gives high penalization to the misclassification of the samples in the minority class. But as we mentioned above, the misclassification cost is often unknown.

To address the imbalanced classification problem without using any priori knowledge, we design a novel method that reformulates the imbalanced classification problem as a partial ranking problem. First, we partition the given *n* samples 〈(*x*_1_,*y*_1_),⋯,(*x*_*n*_,*y*_*n*_)〉 with *y*_*i*_∈{−1,1} into two parts, *X*=〈(*x*_1_,1),⋯,(*x*_*S*_,1)〉 and $\bar {X} = \langle (\bar {x}_{1},-1), \cdots, (\bar {x}_{T}, -1) \rangle $. The first part contains *S* positive samples (minority class) and the second part *T*=*n*−*S* negative samples (majority class). We construct a training set *Z*=〈*z*_1_,⋯,*z*_*K*_〉 from *X* and $\bar {X}$, where a data point $z_{k} = (x_{k}, \bar {x}_{k})$ consists of a sample *x*_*k*_∈*X* and a sample $\bar {x}_{k} \in \bar {X}$. Suppose *F* denotes a function $F(x)\in \mathcal {R}$. We define an indicator function on the training set *Z* as

(2) $$ \mathrm{I}(z_{k}) = \left\{ \begin{aligned} 0 & \qquad F(x_{k}) \geq F(\bar{x}_{k})\\ 1 & \qquad F(x_{k}) < F(\bar{x}_{k}) \end{aligned} \right.  $$

Our target is to find a scoring function that can minimize the following loss function

(3) $$ L(F)= \sum\limits_{k=1}^{K} \mathrm{I}(z_{k}).   $$

Minimizing Eq. (3) with respect to *F* is to solve a combinatorial problem and often intractable. The traditional work-around is either to look for an approximate solution using a greedy algorithm, or to resort to a convex relaxation. Here we relax Eq. (3) and get the following function

(4) $$ \widetilde{L}(F)= \frac{1}{2}\sum\limits_{k=1}^{K}{\left(\max\!\left\{0,F(\bar{x}_{k})-F(x_{k})+\tau\right\}\right)^{2}},  $$

where *τ* is a scalar that is used to avoid the trivial solutions (making *F* as a constant). We may choose the absolute function instead of the square function but the absolute function is not continuous at changing point, which complicates the optimization process. Our goal is to find a function *F* that minimizes $\widetilde {L}(F)$.

### **Proposition1**.

$\widetilde {L}(F)$** is convex.**

### *Proof*.

Because *max*(0,.)≥0, the square of *max*(0,.) is non-decreasing. Because $F(\bar {x}_{k})-F(x_{k})+\tau $ is an affine function of *F* and *max*(0,.) is pointwise supremum (maximum), $\max \!\left \{0,F(\bar {x}_{k})-F(x_{k})+\tau \right \}$ is convex. Therefore, $\widetilde {L}(F)$ is convex.

The function *F* can be any type of functions. In our approach, we consider the function *F* as a sum of multiple base functions,

(5) $$ F(x) = \sum\limits_{m=1}^{M} f_{i}(x).  $$

The direct way to find *F*(*x*) is the gradient boosting approach that starts with the function *f*_0_(*x*)=0 and iteratively adds base functions *f*_*i*_(*x*) to minimize the loss function *L*(*F*). In each iteration, we set as target values the negative gradient of the loss function *L*(*F*) with respect to *F*. Let *F*_*m*−1_ denote the sum of *m*−1 base learners. For a data point $z_{k} = (x_{k}, \bar {x}_{k})$, the negative gradients evaluated at *F*=*F*_*m*−1_ are:

(6) $$ { \small\begin{aligned} r^{m}_{x_{k}}=&-\frac{\partial{\widetilde{L}(F)}}{\partial{F(x_{k})}}|_{F=F_{m-1}}\\ =&\left\{\!\! \begin{array}{ll} F_{m-1}(\bar{x}_{k})-F_{m-1}(x_{k})+\tau&\;\text{if}\; F_{m-1}(x_{k})<F_{m-1}(\bar{x}_{k})+\tau \\ 0&\;\;\text{otherwise} \end{array} \right.\\ r^{m}_{\bar{x}_{k}}=&-\frac{\partial{\widetilde{L}(F)}}{\partial{F(\bar{x}_{k})}}|_{F=F_{m-1}}\\ =&\left\{\!\! \begin{array}{ll} F_{m-1}(x_{k})-F_{m-1}(\bar{x}_{k})-\tau&\;\text{if}\; F_{m-1}(x_{k})<F_{m-1}(\bar{x}_{k})+\tau \\ 0&\;\;\text{otherwise} \end{array} \right. \end{aligned}}  $$

□

**Figure 1:**
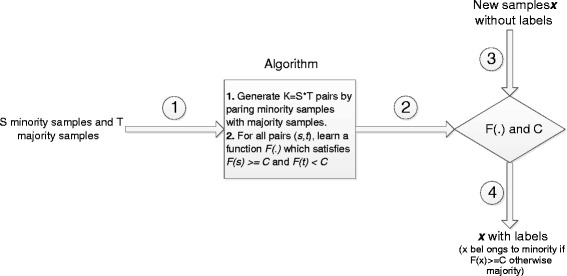
**The training and testing of our method in real applications.**

We choose the regression tree as the base function to fit the negative gradient $r^{m}_{x_{k}}$ and $r^{m}_{\bar {x}_{k}}$ with respect to *x*_*k*_ and $\bar {x}_{k}$, respectively. If the learned regression tree closely matches the target value, adding it with a multiplier *ρ* to the additive model will decrease the loss. The whole gradient boosting procedure for learning the function *F*(*x*) is described as follows:



Figure 1 illustrate how to apply our algorithm in real applications for training and testing (or predicting). Suppose the training data for the training of our algorithm contains *S* minority samples and *T* majority samples, the algorithm first builds a new data sets containing *K*=*S*×*T* pairs by pairing minority samples with majority samples, and next learns a function  and a cut-off threshold *C* for all pairs (*s*,*t*), which satisfies *F*(*s*)≥*C* and *F*(*t*)<*C*.

The learned function  shall separate the training samples as much as possible. For each new sample *x* without class labels, we first compute the value *F*(*x*) and then assign *x* as minority if *F*(*x*)≥*C* or majority if otherwise. In this work, we choose *C* as the middle point between the average of *F* values of positive samples and the average of *F* values of negative samples.

(7) $$ C = \frac{\frac{1}{S}\sum_{s=1}^{S}F(x_{s}) + \frac{1}{T}\sum_{t=1}^{T}F(\bar{x}_{t})}{2}  $$

We name our method “RankCost” as the goal of this method is to find a partial ranking function *F* to replace the predefined cost matrix to solve imbalanced classification problem. To evaluate the performance of RankCost in medical decision making, we compare it with AdaBoost [27], AdaCost [21], and Cost-sensitive decision tree [18].

### Data preparation

Four medical diagnosis data sets are obtained from UCI machine learning repository [29] for the tests. All four data sets are publicly available. These four data sets are from four different disease studies, which are breast cancer, hepatitis, diabetes and sick euthyroid. All of them have binary labels, one for the abnormal category (positive cases) and the other the normal category (negative cases). A brief summary of these four data sets is provided in Table 1.

**Table 1:** **Data set summary**

**Data set**	**Attributes**	**Positives (P)**	**Negatives (N)**	**Ratio (P/N)**
Breast Cancer	9	85	201	0.423
Hepatitis	19	32	123	0.260
Diabetes	8	268	500	0.536
Sick Euthyroid	25	293	2870	0.102

#### Breast cancer data

The breast cancer data was released by the University Medical Centre, Institute of Oncology, Ljubljana, Yugoslavia. Each instance is described by 9 attributes, 3 of which are linear and 6 are nominal. There are 286 instances in this data set, 9 instances with missing values. Class distributions are 29.7% of recurrence-events (positive class) and 70.3% of no-recurrence-events (negative class).

#### Hepatitis data

The second data is from a study of hepatitis, which includes only 155 instances in the whole data set. Each instance is described by 19 attributes with only one being continuously valued. The data set is composed of 32 positive instances (20.65%) in class “DIE” and 123 negative instances (79.35%) in class “LIVE”.

#### Diabetes data

The third data set is from a study of diabetes in Pima Indian population. Each sample is described by 8 continuously valued attributes. 268 samples were identified as positive and the other 500 samples were identified as negative. The two classes are non-evenly distributed with 34.9% of positive instances and 65.1% of negative instances, respectively.

#### Sick Euthyroid data

The fourth data set is from a study of euthyroid sick. The data were collected with 25 attributes, 7 being continuous and 18 being Boolean values. The data set contains 3,163 instances, with 9.26% of the instances being euthyroid and the remaining 90.74% being negative. There are several instances with missing attribute values.

### Performance evaluation

In an imbalanced classification problem, the minority class is often referred to as the positive class and the other one as the negative class. Samples can be categorized into four groups after a classification process, which is denoted in the confusion matrix presented in Table 2. Since the sample in the positive class has the high identification importance, we only evaluate our approach based on the performance of the positive class. In general, there are two well-accepted measures: True Positive Rate and Positive Predictive Value. True Positive Rate (TPR) is defined as

(8) $$ TPR = \frac{TP}{TP+FN}.  $$

**Table 2:** **Confusion matrix table**

	**Predicted as positive**	**Predicted as negative**
Actually positive	True positive (TP)	False negative (FN)
Actually negative	False negative (FP)	True negative (TN)

Positive Predictive Value is defined as

(9) $$ PPV = \frac{TP}{TP+FP}.  $$

To balance these two measures, F-measure is suggested in [30], which is defined as

(10) $$ F-measure = \frac{(1+\beta^{2})\times TPR \times PPV}{\beta^{2}\times TPR + PPV},  $$

where *β* corresponds to the relative importance of TPR versus PPV and it is typically set to 1. The F-measure incorporates TPR and PPV into a single number. It basically represents a harmonic mean between them. It follows that the F-measure is high when both TPR and PPV are high [31]. This indicates that F-measure is able to evaluate the performance of a learning algorithm on the class of our interest.

To evaluate our proposed method RankCost, we specially select three well-known methods to compare, which are AdaBoost [27], AdaCost [21], and Cost-sensitive decision tree [18]. AdaBoost is chosen for the reason that it also does not require a priori cost in handling imbalanced data classification. AdaCost is a cost-sensitive variant of AdaBoost, which requires a priori cost to adjust the weights of samples in different classes. Cost-sensitive decision tree is a popular cost-sensitive classifier for imbalanced classification problems. However, as we mentioned above, the misclassification costs are often unknown in medical decision making and the performances of cost-sensitive classifiers may vary significantly to different misclassification costs. Therefore, in our experiments, we first test AdaCost and Cost-sensitive decision tree on various cost settings and then choose the cost settings with which AdaCOST and Cost-sensitive decision tree can achieve the best performance.

All experiments are performed by following the standard practice of 10-fold cross validation. Each data set is split into ten disjoint subsets using random sampling. Nine of them are used to train the model and the remaining one is used to test the model. This procedure is repeated 10 times so that each partition is used as the test data once. All four methods use exactly the same ten testing and validation data sets, each of which is 10*%* of the entire data. The results for each method are the average of the 10-fold cross-validation. Regarding the cross validation in our experiments, not only is the coefficient (or weight) of each predictor cross-validated, but also the selection of the predictors is also cross-validated. The cost settings for AdaCost and Cost-sensitive decision tree is chosen from the set [ 1.0:0.1,1.0:0.2,1.0:0.3,1.0:0.4,1.0:0.5,1.0:0.6,1.0:0.7,1.0:0.8,1.0:0.9]. The cost of misclassifying a minority sample as a majority sample is always set 1.0. The cost of misclassifying a majority sample as a minority sample is set from 0.1 to 0.9.

## Results

**Figure 2:**
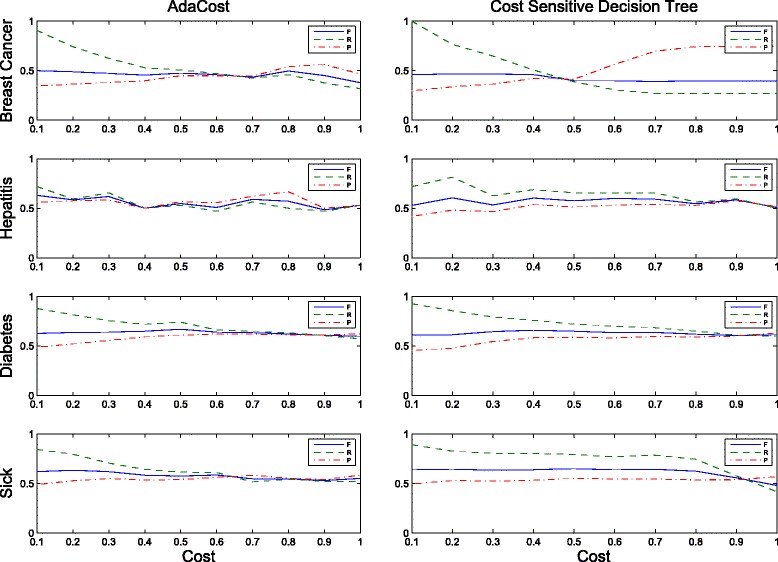
**F-measure, TPR(R) and PPV(P) values of AdaCost and Cost-sensitive decision tree with respect to the different cost settings on four medical data sets.**

Figure 2 shows the F-measure (F), TPR (R), and PPV (P) values of minority class of AdaCost and Cost-sensitive decision tree with respect to the different cost settings on four medical data sets. We can see that in the test on the hepatitis data set (the second row in Figure 2), the performances of both methods fluctuate noticeably from one setting to another setting. The highest values of three measures for AdaCost are 0.628 (F), 0.719 (R), and 0.667(P), and the lowest values are 0.484 (F), 0.469 (R), and 0.500 (P). For cost-sensitive decision tree, the highest values are 0.603 (F), 0.813 (R), and 0.576(P), and the lowest values are 0.508 (F), 0.500 (R), and 0.418 (P). One possible explanation for the high variances in the performances of both methods is that the number of samples in the hepatitis data set is not big enough to learn a stable model with respect to the number of attributes. Therefore, the performance of these two methods may vary a lot across different cost settings. In this situation, it is very difficult to select an appropriate cost in medical decision making. In the other three tests, the F-measure values of these two methods are quite constant. However, the TPR and PPV values still have a large variation. To make comparison between our method and these two cost-sensitive methods, we select the cost settings with which both cost-sensitive methods have the best F-measure values.

Table 3 summarizes the performance comparison among AdaBoost, Cost-sensitive decision tree, AdaCost, and our method RankCost with respect to three measures and their 95*%* confidence intervals. The results shown in Table 3 indicate that in terms of F-measure, RankCost performs equally well with cost-sensitive methods on all four medical data sets. In terms of TPR, it performs better in three data sets. Compared to AdaBoost, our method performs better in all experiments. AdaBoost fails in the test on the sick euthyroid data set. The reason is because the class ratio of minority to majority is very low (10.2*%*). This result justifies the conjecture that AdaBoost may fail on extremely imbalanced data sets because its goal is to maximize the overall prediction accuracy.

**Table 3:** **Performance comparison among AdaBoost, Cost-sensitive decision tree, AdaCost, and our method RankCost with respect to F-measure (F), TPR (R), and PPV (P) values and and their 95** ***%*** **confidence intervals**

		**AdaBoost**	**Cost-sensitive**	**AdaCost**	**RankCost**
			**Decision tree**		
Breast Cancer	Cost		1:0.2	1:0.1	
	F	0.465 [0.217,0.713]	0.468 [0.366,0.570]	0.502 [0.358,0.646]	0.494 [0.376, 0.612]
	R	0.388 [0.104, 0.672]	0.765 [0.423,1.107]	0.906 [0.603, 1.209]	0.494 [0.270, 0.718]
	P	0.579 [0.152, 1.000]	0.337 [0.263, 0.411]	0.347 [0.211, 0.483]	0.494 [0.392, 0.596]
Hepatitis	Cost		1:0.4	1:0.1	
	F	0.5 [0.002,0.998]	0.603 [0.261, 0.945]	0.628 [0.200,1.056]	0.628 [0.280,0.976]
	R	0.469 [-.215, 1.153]	0.688 [0.212, 1.164]	0.719 [0.165,1.273]	0.843 [0.465,1.221]
	P	0.536 [-.122,1.194]	0.537 [0.047,1.022]	0.561 [0.159, 0.963]	0.500 [0.158,0.842]
Diabetes	Cost		1:0.4	1:0.5	
	F	0.595 [0.447,0.743]	0.658 [0.472,0.844]	0.661 [0.493,0.829]	0.692 [0.532,0.852]
	R	0.526 [0.308,0.744]	0.757 [0.589,0.825]	0.731 [0.527,0.935]	0.802 [0.638,0.966]
	P	0.684 [0.508,0.860]	0.582 [0.400,0.764]	0.603 [0.403,0,803]	0.609 [0.431,0.787]
Sick Euthyroid	Cost		1:0.2	1:0.2	
	F	0.007 [-.033,0.047]	0.645 [0.549,0.741]	0.616 [0.538,0.694]	0.612 [0.538,0.686]
	R	0.003 [-.017,0.023]	0.826 [0.714,0.938]	0.785 [0.659,0.911]	0.942 [0.814,1.070]
	P	0.1 [-.500,0.700]	0.53 [0.416,0.644]	0.507 [0.429,0.585]	0.453 [0.389,0.517]

The cost settings are those with which AdaBoost and Cost-sensitive decision tree can achieve the best F-measure values.

In our experiments, we observe that the results on hepatitis data show high variance. The main reason is due to the number of attributes. There are 19 attributes in the hepatitis data, which requires a much large data set in order to train a reliable and consistent model across the multiple runs of validation. However, we only have 155 samples in total. In such a situation, the literature suggested evaluation method is the leave-one-out cross-validation, in which the test data only contains one sample and all the others are used in the training. The number of runs (or folds) is equal to the number of samples. However, in the evaluation using hepatitis data, adding a few more samples in the training data is still far from enough to train a stable model. Furthermore, there are some critical issue in leave-one-out cross-validation. Besides the low efficiency. The major one is that each run is highly correlated with the others. That correlation may lead to the significant underestimation of the variance when the trained model is applied to new data because most of the trained models in leave-one-out evaluation will be nearly identical. Therefore, the trained model from the leave-one-out cross validation is very prone to over-fitting. Taking all these issues into consideration, we eventually choose the most popular one, which is 10 cross-validation.

### Convergence of RankCost

**Figure 3:**
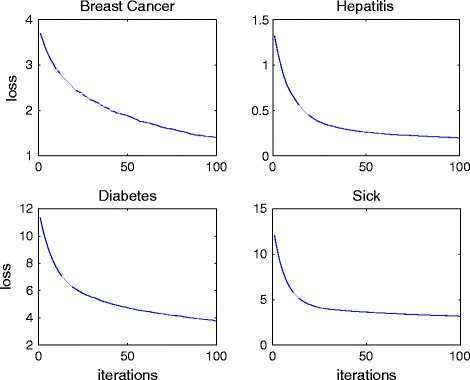
**The curve of loss function in RankCost on four data sets.**

To show the convergence of RankCost, the values of loss function during the learning process on four data sets are collected and presented in Figure 3. First, we can empirically conclude that the loss function defined in Eq. (4) is convex. Second, we can observe that the convergence speed is fast because the value of the loss function drops very quickly in the first few iterations and the learning process can reach the optimal status in around one hundred iterations.

## Discussion and conclusions

In medical data analysis, it often happens that data are composed of a minority (positive or abnormal) group and a majority (negative or normal) group and the cost of misclassifying a minority sample as a majority sample is highly expensive. It is a challenging task to learn an effective classification model based on imbalanced data. The traditional approaches often use a priori cost to adjust the learning process in the pursuit of optimal classification function. However, this priori cost is often unknown and hard to estimate in medical decision making. This work presents a novel approach, namely RankCost, for learning from medical imbalanced data sets without using a priori cost. In RankCost, the traditional imbalanced classification problem is reformulated into a partial ranking problem. Instead of focusing on the class prediction accuracy, RankCost is to learn a non-parametric scoring function which can maximize the difference between the minority class and the majority class. The boosting technique is adopted in RankCost to learn the scoring function, and the relative importance of the minority class over the majority class is naturally reflected in the learning process. The performance of RankCost is illustrated by tests on four medical data sets varying in size, dimension, and imbalanced ratio. The experimental results obtained indicate that our approach achieves comparable performance against two cost-sensitive methods and outperforms the non-cost-sensitive method AdaBoost. Importantly, our approach does not require any priori knowledge, which makes our method more practical in medical decision making.

There are some limitations in our works. First, our approach does sacrifice the performance of the majority class for the minority class since it only aims to improve the prediction accuracy of the minority class. In medical decision making, misclassifying a majority sample as a minority sample is also a serious issue in some situations. Second, our approach can only handle two class classification at this moment. Multi-class imbalanced learning problems are also very popular and very difficult to solve in medical decision making. Our future research will address these issues by considering different types of scoring functions.
